# Event and Comment

**Published:** 1914-06-15

**Authors:** 


### DENTAL REGISTER.

**Vol. LXVIII. June 15, 1914. No. 6**

### EVENT AND COMMENT.

NEW SECTION OF THE NATIONAL DENTAL ASSOCIATION. At the last meeting of the National Association in Kansas City, a new organization was perfected and has been made a special section. It is to be constituted of the officers of the various State Societies which are members of the National. Its business will be to hear reports from the several State Societies as to special methods adopted for increasing the interest, and for making more valuable the programmes. Such an organization ought to be immensely helpful in passing around those things which have been found helpful in not only the clinics and papers, but there are many business or executive matters that could be distributed to good advantage. It is this get together idea, that seems to be in the atmosphere these days, that promises great things for the future. If all the dental societies would earnestly take up and consider some one thing, like for instance, the encouragement of scientific research, or the wisdom of increasing the efficiency of dental instruction in our colleges, or the unification of our dental laws: a sentiment would be created that would soon spread throughout the land, and by general consensus of opinion we would take such steps as might be necessary to put into effect the sentiment of the profession on these important matters.

NEW YORK MEDICAL SOCIETY. A report of this society just published in its journal, lists a membership of over 7000, which is more than four times the membership of any dental society and until the present time more than our National membership. The dues are three dollars per member, yielding more than $21,000. With this money it publishes its own medical journal, which is a most excellent one, at an expense of nearly $9,000, and a directory of its members at an expense of as much more, which latter it seems to us is unnecessary in view of the fact that the A. M. A. publishes a good directory of physicians for the entire country. It however, receives some returns from the sale of its directories, and also from the advertising in the medical journal. It spent also last year nearly $5,000 for legal expenses in defending its members in suits for malpractice. It also paid nearly $700 for delegates expenses to the American Medical and other Association meetings. It is a live organization and some of its methods might be adopted by our State Dental Societies with good results.

NEW DEAN FOR CALIFORNIA. Dr. Jas. G. Sharp, the Dean of the Dental School of the University of California, has resigned, and Dr. Guy S. Millberry, formerly secretary of the school succeeds to the office. Dr. Sharp will retain his teaching position in the faculty. Dr. Millberry is a fine executive officer and he will prove a worthy successor to Dr. Sharp in this position.

OHIO'S CONTRIBUTION TO THE NATIONAL. The State of Ohio has placed its entire membership of 1077 in the re-organized National Dental Association. Next to Illinois, this is the largest component so far affi.iated. New York will no doubt in time out-distance Ohio and perhaps Illinois. These three states will have the largest representation in the House of Delegates, and consequently be important factors in political affairs. Their usefulness in other directions will also be important as many of our most active workers are to be found in these States. It is gratifying to see the unanimity with which the re-organization scheme is being adopted by the several States. It looks as though it will soon be unanimous. It is coming so easy that the best friends of the scheme are beginning to feel uneasy lest they shall be unable to carry out all the promised advantages they have been holding out. In union there is strength and in members there is always enthusiasm and we believe there will be a wonderful power for good.

DENTISTRY IN THE POLAR REGIONS. The Crocker Land Expedition which is sailing for a three years survey of the Polar lands has been supplied with an outfit of dental instruments by the National Dental Association, and the medical surgeon has been trained to use them at least to the extent of rendering such temporary service as may become necessary to the members of the expedition. If a base ball club needs a special dental surgeon, why should not one be attached to such an expedition? If Mr. Roosevelt had taken a dentist with him on his South American trip, we might have some fine crania for our museums.

"GOOD HEARING." We took occasion in the March issue to comment on an editorial statement in the *New Jersey Dental Journal*, not with the intention of criticising the intent or content of the editorial, as the editor evidently supposed judging from the nature of his reply; but with the hope that the sentiment he there expressed might be true and that the star of hope was again emerging after a long sleep in that part of our country from which we used to get inspiration, and sure enough we struck fire, for the editor comes back with a fine showing of real spirit. We are not so sure of his conclusions, as we have watched this problem for many years, and we are in fairly close touch with its workings at the present time. We really enjoy his enthusiasm and optimism and we regret that we can not join with him and his cohorts in accomplishing the splendid things that he so confidently expects to see done, and right away too. He thinks the "up and doing dental schools are seriously contemplating a four years course." Yes, we happen to know that a few schools are seriously considering this matter; in fact the seven schools that are members of the Dental Faculties Association of American Universities have passed a resolution asking their respective governing bodies to establish a four years course in dentistry, and we confidently expect that it will be done in these seven schools in the next year or so. The National Dental Association and the National Association of Examiners and various State and Local Societies will doubtless endorse the proposition. So will all our editors and most practitioners, but will all the schools adopt it even though the sentiment is as strong as we think it to be because of such endorsement as we have here indicated?

A few years ago a test on this matter was had and all our schools adopted the four year course for one year, and because it came near financially bankrupting a few schools and all the others were so seriously affected financially, there was a double quick retrograde movement back to the three years course, before the matter had been given a fair trial. It will be difficult to induce these same schools to try this thing again. It will take more argument than all our editors and dental organizations combined can muster to convince some of these schools of the practicability of such a scheme.

Our editor also thinks that another important and practicable scheme is to force the education of the graduate even after he leaves college by means of the dental society and the dental journals. Both of these instrumentalities have been used in the past to most excellent purpose in this connection, and no doubt they will be important factors in the future development of professional attainments. But we can't help again referring to the fact that only a small per cent of our profession attend dental meetings consistently, and about the same proportion, and more than likely the same men, read our journals. It is the claim of the officers that the New National Dental Association will have a membership of 15,000 at the time of the annual meeting in July. Does any one suppose there will be an attendance of one third or

5000 members at that meeting? It may be true that all the 15,000 will have the printed proceedings and may be they will read them. The dental journal claiming the largest circulation of paid subscribers claims to have nearly 15,000 subscribers, and these were secured at considerable cost and much extraordinary effort on the part of the publishers, and it is safe to say that the larger number of regular subscribers to any journal take only one or two journals at most, and the probabilities are that not more than three or four paid for dental journals have a subscription list of over six thousand bona fide subscribers. How then shall we expect to reach adequately the masses of our profession who will not attend our society meetings, or read our literature, or in fact take any real professional interest in dentistry expect as a means of securing a living? Now we are not taking a pessimistic view of this matter at all; we believe in the profession because we know its value to humanity and we confidently expect it to rank with other noble profession, if not now, in the near future when its real worth becomes known to the general public, and we know of no better way of hastening the coming of our kingdom than along the lines suggested, but we have our moments of mis-givings, the result of apparent indifference on the part of members of our profession who know what should be done and yet because of indifference or selfish interests involved, refuse to help with their cordial and sincere co-operation.

This is why we challenged our editorial confrerre and his statement, we believe he has the right sentiment and his ideals are correct; but we would like to know if he represents his constituency, or whether he has only had a fine dream that he and every other true professional man wishes might be true.

